# Bone marrow and peripheral blood expression of *ID1* in human gastric carcinoma patients is a *bona fide* indicator of lymph node and peritoneal metastasis

**DOI:** 10.1038/sj.bjc.6605085

**Published:** 2009-06-02

**Authors:** M Iwatsuki, T Fukagawa, K Mimori, H Nakanishi, S Ito, H Ishii, T Yokobori, M Sasako, H Baba, M Mori

**Affiliations:** 1 Department of Surgical Oncology, Medical Institute of Bioregulation, Kyushu University, 4546 Tsurumihara, Beppu 874-0838, Japan; 2 Department of Gastroenterological Surgery, Graduate School of Medical Sciences, Kumamoto University, 1-1-1 Honjo, Kumamoto 860-8556, Japan; 3 Gastric Surgery Division, National Cancer Center Hospital, 5-1-1 Tsukiji, Chuo-ku, Tokyo 104-0045, Japan; 4 Division of Oncological Pathology, Aichi Cancer Center Research Institute, 1-1 Kanokoden, Chikusa-ku, Nagoya 464-8681, Japan; 5 Department of Gastroenterological Surgery, Aichi Cancer Center Central Hospital, 1-1 Kanokoden, Chikusa-ku, Nagoya 464-8681, Japan

**Keywords:** *ID1*, gastric cancer, bone marrow, peripheral blood, lymph node metastasis, peritoneal dissemination

## Abstract

Recent studies have showed that the bone marrow-derived endothelial progenitor cells play critical roles in metastasis and that *ID1* is required in metastasis as regulator of angiogenesis. Therefore, we investigated the clinical significance of *ID1* mRNA expression in bone marrow and peripheral samples in patients with gastric cancer. Two hundred and eighty-nine bone marrow and 196 peripheral blood samples from gastric cancer patients were collected and analysed by quantitative RT–PCR for *ID1*. The ID1 protein expression in one bone marrow, three metastatic lymph nodes and three peritoneal disseminated tumours was examined by immunohistochemical methods. In both bone marrow and peripheral blood samples, *ID1* mRNA expression in the metastatic group was significantly higher than in any other group (*P*=0.003, *P*=0.0001, respectively) and significantly associated with lymph node metastasis and peritoneal dissemination. The cells in bone marrow with metastatic cancer stained strongly with ID1 compared with those of healthy volunteers. The expression of *ID1* mRNA in bone marrow and peripheral blood was significantly associated with lymph node metastasis and peritoneal dissemination, and therefore constitutes a predictable marker for lymph node metastasis and peritoneal dissemination.

The presence of isolated tumour cells (ITCs) is an important factor in the metastasis of solid cancers. Recently, we investigated the presence of ITCs in peripheral blood and in bone marrow, using quantitative RT–PCR in more than 800 cases of gastric cancer (Mimori *et al*, 2008). We found that ITCs circulate in a range of clinical stages of gastric cancer. These data suggested that host cells might play a supportive role for metastasis.

Recently, Kaplan *et al* reported that bone marrow-derived haematopoietic progenitor cells that express vascular endothelial growth factor receptor 1 (VEGFR-1) home to tumour-specific pre-metastatic sites and form cellular clusters before the arrival of tumour cells (Kaplan *et al*, 2005). In a large-scale study of gastric cancer cases, we recently reported that the simultaneous expression of ITC-associated genes and high levels of expression of *VEGFR-1* in bone marrow were significantly associated with haematogeneous metastases (Mimori *et al*, 2008). Gao *et al* determined that the bone marrow-derived endothelial progenitor cells (EPCs) were critical regulators of angiogenic switching (Gao *et al*, 2008). Furthermore, they showed that tumours induce expression of *ID1* in EPCs and that suppression of *ID1* after metastatic colonisation blocked EPC mobilisation, inhibited angiogenesis and impaired pulmonary macrometastases. ID proteins are inhibitors of DNA binding of basic helix-loop-helix (bHLH) transcription factors by heterodimerisation with the bHLH proteins (Benezra *et al*, 1990). *ID1* has been reported to be associated with the undifferentiation of cancer cells, severe malignant grade of tumour, invasion of tumours and worse prognosis in several tumours (Fong *et al*, 2003).

In this study, we investigated the clinical significance of the *ID1* mRNA expression in bone marrow and peripheral blood samples obtained from gastric cancer patients. The results showed that the *ID1* mRNA expression in bone marrow and peripheral blood was significantly associated with lymph node metastasis and peritoneal dissemination. Thus, *ID1* is a *bona fide* predictive marker for both pathologic parameters, each of which is an established definitive prognostic indicator in gastric cancer.

## Materials and methods

### Patients

Physicians (TF and MS) collected bone marrow and peripheral blood samples from 289 Japanese gastric cancer patients who underwent surgery from 2001 to 2004 at the Central Hospital, the National Cancer Center, Tokyo, Japan. The documented informed consent was obtained from all patients and the protocol of the study was approved by the local ethics committee. There were 190 male and 99 female patients with an average age of 62.3 and a range of 24–86 years (Table 1). Seventy of the patients showed peritoneal dissemination at the time of surgery or at postoperative follow-up. Among the 289 cases, 76, 60, 62 and 91 were classified as stages I, II, III or IV, respectively, according to the Treaty for Japanese Gastric Cancer Association (Maruyama *et al*, 2006).

### Bone marrow and peripheral blood samples from gastric cancer patients

Aspiration of both bone marrow and peripheral blood was conducted under general anaesthesia immediately before surgery as described earlier (Mimori *et al*, 2008). The bone marrow aspirate was obtained from the sternum using a bone marrow aspiration needle and peripheral blood was obtained through a venous catheter. The first 1.0 ml of bone marrow and peripheral blood were discarded to avoid contamination by the skin. The second collected 1.0 ml of bone marrow and peripheral blood were put into 4.0 ml of Isogen-LS (Nippon Gene, Toyama, Japan) and stored at −80 °C until RNA extraction.

### Total RNA extraction and first-strand cDNA synthesis

Samples transferred from Tokyo to Beppu remained frozen while in transit. Total RNA was extracted from bone marrow and peripheral blood according to the manufacturer's protocol as described elsewhere (Iinuma *et al*, 2006). The reverse transcriptase reaction (RT) was performed as described earlier (Mori *et al*, 1995). The first-strand cDNA was synthesised from 2.7 *μ*g of total RNA in 30 *μ*l reaction mixtures containing 5 *μ*l 5 × RT buffer (BRL, Gaithersburg, MD, USA), 200 *μ*M dNTP, a 100 *μ*M solution of a random hexadeoxynucleotide mixture, 50 units of Rnasin (Promega, Madison, WI, USA), 2 *μ*l of 0.1 M dithiothreitol and 100 units of Maloney leukemia virus RT (BRL). The mixture was incubated at 37°C for 60 min, heated to 95°C for 10 min and then chilled on ice.

### Quantitative real-time RT–PCR

The sequences of *ID1* mRNA were as follows: sense, 5′-CCAGTGGCAGCACCGCCACC-3′, and anti-sense, 5′-CGGATTCCGAGTTCAGCTCC-3′. We used glyceraldehyde-3-phosphate-dehydrogenase (*GAPDH*) as an internal control. The primers were as follows: sense, 5′-TTGGTATCGTGGAAGGACTCTA-3′, and anti-sense, 5′-TGTCATATTTGGCAGGTT-3′. Real-time monitoring of PCR reactions was performed using the LightCycler system (Roche Applied Science, Indianapolis, IN, USA) and SYBER-Green I dye (Roche Diagnostics, Tokyo, Japan) to detect *ID1* in bone marrow and peripheral blood. Monitoring was performed according to the manufacturer's instructions, as described earlier (Ogawa *et al*, 2005). In brief, a master mixture was prepared on ice, containing 1 *μ*l of cDNA, 2 *μ*l of DNA Master SYBER-Green I mix, 50 ng of primers and 2.4 *μ*l of 25 mM MgCl_2_. The final volume was adjusted to 20 *μ*l with water. After the reaction mixture was loaded into glass capillary tubes, quantitative RT–PCR was performed with the following cycling conditions: initial denaturation at 95°C for 10 min, followed by 40 cycles of 95°C for 10 s, annealing at 62°C for 10 s and extension at 72°C for 10 s. After amplification, products were subjected to a temperature gradient from 67°C to 95°C at 0.2°C s^−1^, under continuous fluorescence monitoring, to produce a melting curve of products.

### Data analysis for RT–PCR

We used the LightCycler Software version 3.5 program (Roche Molecular Biochemicals, Basel, Switzerland) to calculate the cycle numbers. After proportional baseline adjustment, a fit point method was used to determine the cycle in which the log-linear signal was first distinguishable from the baseline. This cycle number was used as the crossing point value. A standard curve was produced by measuring the crossing point of each standard value and plotting it against the logarithmic value of the concentration. Concentrations of unknown samples were calculated by plotting their crossing points against the standard curve and dividing by *GAPDH* content. The results of RT–PCR were sent from Beppu to Tokyo for analyses.

### Immunohistochemistry

Immunohistochemistry was performed on paraffin-embedded specimens obtained from patients with metastatic gastric cancer and two healthy volunteers. Tissue sections were deparaffinised, soaked in 0.01 M sodium citrate buffer and boiled in a microwave for 5 min at 500 W to retrieve cell antigens. The primary rabbit polyclonal antibody against ID1 (C-20; Santa Cruz Biotechnology, Santa Cruz, CA, USA), which detects ID1 in paraffin-embedded human tissue sections and does not crossreact with ID2, ID3 or ID4 (Maruyama *et al*, 1999), was used at a dilution of 1 : 100. The blocking peptide to ID1 (sc-488P, Santa Cruz Biotechnology) was used as a negative control (Supplementary Figure 4). Tissue sections were immunohistochemically stained using the avidin–biotin-peroxidase method (LSAB+ system-HRP; DAKO, Kyoto, Japan). All sections were counterstained with haematoxylin.

### Statistical analysis

The expression of *ID1* was adjusted in each case for *GAPDH* expression. For continuous variables, data were expressed as the means±s.d. The relationship between *ID1* mRNA expression and clinicopathlogical factors was analysed using a *χ*^2^ test and Student's *t*-test. All tests were analysed using JMP software (SAS Institute Inc., Cary, NC, USA) and the findings were considered significant when the *P*-value was <0.05.

## Results

### Expression of *ID1* mRNA in bone marrow of gastric cancer

**Figure 1:**
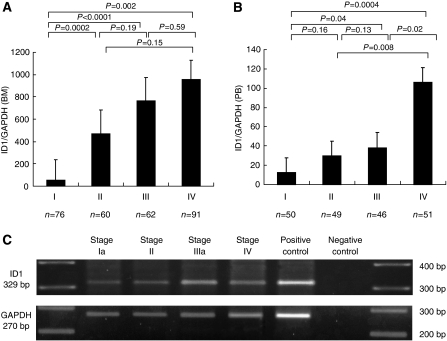
The mean value of *ID1* mRNA expression normalised *GAPDH* in bone marrow (**A**) and peripheral blood (**B**) according to staging classification. Group stage I consisted of patients with tumours that invaded less than the sub-mucosal layer and no lymph node metastasis (BM: *n*=76; PB: *n*=50). Group stage II consisted of patients with tumours that penetrated serosa or lymph node metastasis (Group 1) (BM: *n*=60; PB: *n*=49). Group stage III consisted of patients with tumours invasion of adjacent structures or lymph node metastasis (Group 2 or 3) (BM: *n*=62; PB: *n*=46). Group stage IV consisted of patients with distant metastasis (BM: *n*=91; PB: *n*=51). The mean value of *ID1* mRNA expression in bone marrow and peripheral blood increased along with the progression of stage. The RT–PCR analysis of four representative bone marrow samples was performed in each stage (**C**: upper; *ID1* product size 329 bp, lower; *GAPDH* product size 270 bp).

Figure 1A shows expression of *ID1* mRNA in bone marrow according to staging classification. In bone marrow, the mean expression level of *ID1* mRNA in stage IV (957±169) was significantly higher than other stages (*P*=0.003). Specifically, the levels of stages I, II and III were 54±185, 472±208, and 767±205, respectively. To confirm the specificity of *ID1*, we performed RT–PCR analysis of six representative cases in each stage, which was very close to the average value (Figure 1C). In addition, sequencing of these transcripts confirmed that it was the product of *ID1* (Supplementary Figure 1).

### Expression of *ID1* mRNA in peripheral blood of gastric cancer

In the peripheral blood samples, there was a significant relationship between the expression level of *ID1* mRNA and the progression of gastric cancer cases (Figure 1B). The mean expression level of *ID1* mRNA in stage IV (105±15) was significantly higher (*P*=0.0001) than stages I, II and III (12.4±15.4, 29.6±15.5, and 38.3±16.0, respectively). In addition, there was a significant correlation between the expression of *ID1* mRNA in bone marrow and peripheral blood (*r*=0.23, *P*=0.002, data not shown).

### *ID1* expression and clinicopathological features of gastric cancer patients

**Figure 2:**
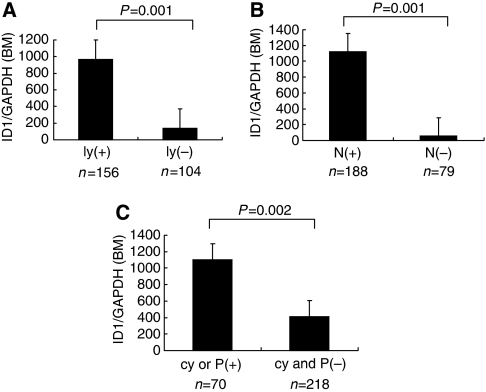
Comparison of the *ID1* mRNA expression in bone marrow of patients with or without lymphatic invasion (ly) (+: *n*=156, −: *n*= 104; **A**), lymph node metastasis (*N*) (+: *n*=188, −: *n*= 79; **B**) and peritoneal cytology (cy) or peritoneal metastasis (P) (+: *n*=70; −: *n*= 218; **C**). In patients with evidence of lymphatic invasion, lymph node metastasis or peritoneal dissemination, the expression of *ID1* mRNA in bone marrow was significantly higher compared to patients without metastasis.

**Figure 3:**
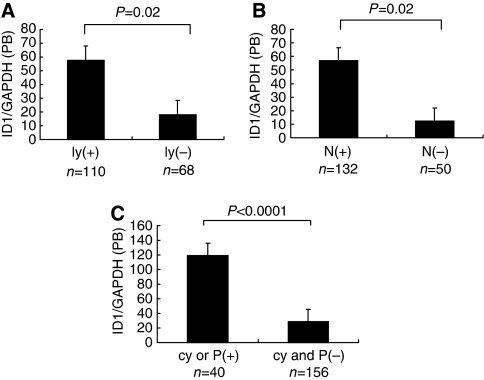
Comparison of the *ID1* mRNA expression in peripheral blood of patients with or without lymphatic invasion (ly) (+: *n*=110, −: *n*=68; **A**), lymph node metastasis (N) (+: *n*=132, -: *n*= 50; **B**) and peritoneal cytology (cy) or peritoneal metastasis (P) (+: *n*=40, −: *n*=156; **C**). In patients with evidence of lymphatic invasion, lymph node metastasis or peritoneal dissemination, the expression of *ID1* mRNA in peripheral blood was significantly higher compared to patients without metastasis.

We examined the clinicopathlogical significance of *ID1* mRNA in samples from bone marrow and peripheral blood (Table 1). In both bone marrow and peripheral blood, there are significant associations with many cliniopathlogical features such as tumour size and depth of tumour invasion. Especially, in patients with evidence of lymphatic invasion, lymph node metastasis or peritoneal dissemination, we found significantly higher expression of *ID1* mRNA in bone marrow samples compared to patients without metastasis. (*P*=0.001, *P*=0.001 and *P*=0.002, respectively, Figures 2A–C). Similarly, in peripheral blood samples, the cases with lymphatic invasion, lymph node metastasis or peritoneal dissemination had significantly higher expression of *ID1* mRNA compared to patients without metastasis. (*P*=0.02, *P*=0.02 and *P*<0.0001 respectively, Figures 3A–C).

### Expression of ID1 protein in bone marrow from patients with metastatic gastric cancer and healthy volunteers

**Figure 4:**
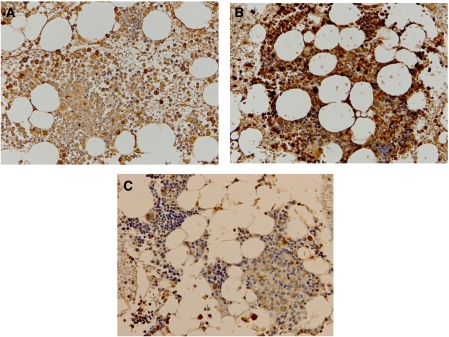
Immunohistochemistry with ID1 antibody, assessing bone marrow from a representative healthy volunteer and metastatic gastric cancer patient. In the bone marrow of healthy volunteer (**A**), the ID1 expression was localised mainly in the nuclei of bone marrow cells. The population of ID1-positive cells in healthy volunteer is lower than that in metastatic patients (**B**). The metastasized cells confirmed to be epithelial cells by HE stain and AE1/AE3 (Supplementary Figures 2A and B) that originated from gastric cancer were stained slightly with ID1 in cytoplasm (**C**). (**A**–**C**: original magnification: × 100).

The ID1 protein expression in bone marrow was evaluated immunohistochemically in studies of metastatic gastric cancer patients and healthy volunteers. In bone marrow of healthy volunteer (Figure 4A), the ID1 expression was localised mainly in the nuclei of bone marrow cells. The population of ID1-positive cells in healthy volunteer is lower than that in metastatic patient (Figure 4B). The ID1 expression of bone marrow cells with metastatic patient was also localised mainly in the nuclei. We also examined the ID1 expression of bone marrow carcinomatosis resulting from gastric cancer. The metastasized cancer cells were confirmed to be epithelial cells by HE (Supplementary Figure 2A) stain and AE1/AE3 (Supplementary Figure 2B). These cells were stained slightly with ID1 antibody in the cytoplasm (Figure 4C).

### ID1 expression in primary lesions and metastatic lesions of gastric cancer

**Figure 5:**
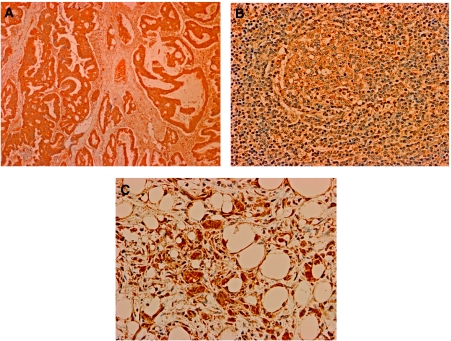
Immunohistochemistry with ID1 antibody assessing primary and metastatic lesions. Most of the primary lesions of gastric cancer were stained strongly with the ID1 antibody. A representative case was shown in (**A**). The ID1 expression was localised in the cytoplasm of cancer cells in primary lesion, metastatic lymph node metastasis (**B**) and peritoneal dissemination (**C**). (Original magnification: **A**: × 40, **B** and **C**: × 100).

We examined the ID1 protein expression immunohistochemically in 30 primary lesions, 3 metastatic lymph nodes and 3 peritoneal disseminated lesions of gastric cancer cases. We found that 20 cases have high ID1 expression in primary lesions (Figure 5A). Some of the cases showed weak (Supplementary Figure 3A) or moderate (Supplementary Figure 3B) ID1 staining. In addition, two of three metastatic lymph nodes and peritoneal disseminated lesions were stained slightly with the ID1 antibody and the ID1 expression was localised in the cytoplasm of cancer cells in primary lesion, metastatic lymph node metastasis and peritoneal dissemination (Figures 5B and C).

## Discussion

Peritoneal dissemination is recognised as the most critical factor in assessing the prognosis of gastric cancer cases (Bando *et al*, 1999). There is no conclusive evidence, however, whether peritoneal dissemination might be established by the lymph node metastasis as well as direct dissemination from the serosal layer of stomach (Yonemura *et al*, 2007). In this study, the *ID1* mRNA expression in bone marrow and peripheral blood was significantly associated with lymph node metastasis and peritoneal dissemination. Therefore, we suggest that peritoneal dissemination of gastric cancer is mediated through lymph node metastasis combined with the *ID1*-expressing endothelial cells from bone marrow. From a clinical point of view, there are no convincing markers for peritoneal dissemination before surgery. Therefore, it is significant that the *ID1* expression in bone marrow and peripheral blood can be used as a reliable marker before surgery to determine which gastric cancer patients are likely to have peritoneal dissemination mediated through lymph node metastasis.

There are two possible sources of the *ID1*-positive cells: ITC and host cells (such as EPCs, as stated by Gao *et al*). With regard to tumour cells, Tsuchiya *et al* showed that the number and size of peritoneal metastatic nodules formed by ID1 and ID3 double-knockdown gastric cancer cells were reduced in comparison to mock-transfected control cells *in vivo* (Tsuchiya *et al*, 2005). Furthermore, Kim *et al* reported that transgenic mice expressing a thymocyte-specific Id1 gene developed T-cell lymphoma *in vivo* (Kim *et al*, 1999). In addition, overexpression of ID1 in the primary cancer cells relative to normal mucosa has been observed in primary human oesophageal (Hu *et al*, 2001) and colorectal cancers (Wilson *et al*, 2001). Those reports found that the ID1 expression was significantly associated with the differentiation of cells and a poor prognosis. In gastric cancer, Han *et al* found that strong immunohistochemical ID1 expression was associated with poorer differentiation and more aggressive behaviour of tumour cells (Han *et al*, 2004). In this study, we also examined the ID1 expression in primary lesion of gastric cancer cases. We found that two-thirds cases have high ID1 expression in primary lesions (Figure 5A). Furthermore, we showed that the metastasized cancer cells from gastric cancer in bone marrow were slightly stained with ID1 antibody (Figure 4C). These findings suggest that *ID1* may be a potential oncogene. As for the origin of ID1-positive cells in bone marrow, these seem to represent ITCs.

On the other hand, in this study, we present two lines of evidence indicating that the origin of the ID1 expression is from host cells, perhaps originating from bone marrow or peripheral blood. First, immunohistochemical studies showed that the population of ID1-positive cells in healthy volunteer is lower than that in metastatic patients (Figure 4A). Thus, ID1-expressing cells are particularly numerous in the bone marrow in which there are relatively few cancer cells. The current findings may suggest that ID1 is not a component of the aggregated cancer cells in the metastatic lymph nodes and peritoneal disseminated tumours, but instead plays a supportive role for gastric cancer cells to form lymph node metastasis and peritoneal dissemination.

Secondary, Gao *et al* found that Id1 was expressed by EPC positive for VE-cadherin and CD31 in peripheral blood (Gao *et al*, 2008). As we expected, the *ID1* expression in peripheral blood was significantly related to the incidence of peritoneal dissemination. In addition, there were significant association between *ID1* expression in peripheral blood and those in bone marrow from gastric cancer cases. This finding may indicate that the expression of *ID1* in peripheral blood originates from the circulating progenitor cells (CPCs), including EPC and from the bone marrow (Lyden *et al*, 2001). Those results may suggest that the origin of *ID1* expression is not only from cancer cells but also from host cells, such as CPCs in bone marrow and peripheral blood.

In summary, we found that the *ID1* mRNA expression in bone marrow and peripheral blood is a reliable predictive marker for lymph node metastasis and peritoneal dissemination, which indicates a poor prognostic outlook in gastric cancer. In addition, our findings suggest that the *ID1* expression originates from not only the cancer cells but also the host side progenitor cells with the cancer-bearing condition. Therefore, we propose that targeting the *ID1*-expressing cells in the bone marrow and/or peripheral blood after surgery represents a new concept for the treatment and/or prevention of metastasis.

## Figures and Tables

**Table 1:** Clinicopathlogial significance of *ID1* mRNA expression in gastric cancer patients

	**Bone marrow**			**Peripheral blood**		
**Features**	**Number**	***ID1* mRNA expression (mean±s.d.)**	***P*-value**	**Number**	***ID1* mRNA expression (mean±s.d.)**	***P*-value**
Age	289	62.3±11.9	—	196	62.9±12.2	—
						
Sex (M : F)	289	190 : 99	—	196	131 : 65	—
						
*Tumour size*			0.01			0.002
⩽5 cm	126	311±146		85	24.8±12.2	
⩾5 cm	160	795±129		110	64.6±10.7	
						
*Depth of tumour invasion* a			0.01			0.004
m, sm	90	210±171		62	12.9±14.2	
mp, ss, se, si	199	744±115		134	62.8±9.6	
						
*Venous invasion*			0.97			0.007
Positive	72	580±199		50	77.8±15.2	
Negative	188	571±123		128	28.9±9.5	
						
*Lymphatic invasion*			0.001			0.02
Positive	156	850±132		110	57.8±10.3	
Negative	104	158±162		68	18.2±13.1	
						
*Lymph node metastasis*			0.001			0.02
Positive	188	800±121		132	56.7±9.6	
Negative	79	58.7±186		50	12.4±15.6	
						
*Peritoneal dissemination* b			0.002			<0.0001
Positive	70	1102±194		40	119±17.1	
Negative	218	412±110		156	28.6±8.6	

a Tumour invasion of mucosa (m), submucosa (sm), muscularis propria (mp), subserosa (ss), penetration of serosa (se) and invasion of adjacent strucures (si).

b Peritoneal dissemination: peritoneal cytology or metastasis positive.
