# Supplementary figures and images for: Bone marrow and peripheral blood expression of ID1 in human gastric carcinoma patients is a bona fide indicator of lymph node and peritoneal metastasis
Source: Br J Cancer. 2009 Jun 2;100(12):1937–42. doi: 10.1038/sj.bjc.6605085 (PMC2714249; doi:10.1038/sj.bjc.6605085)

## Slide 1
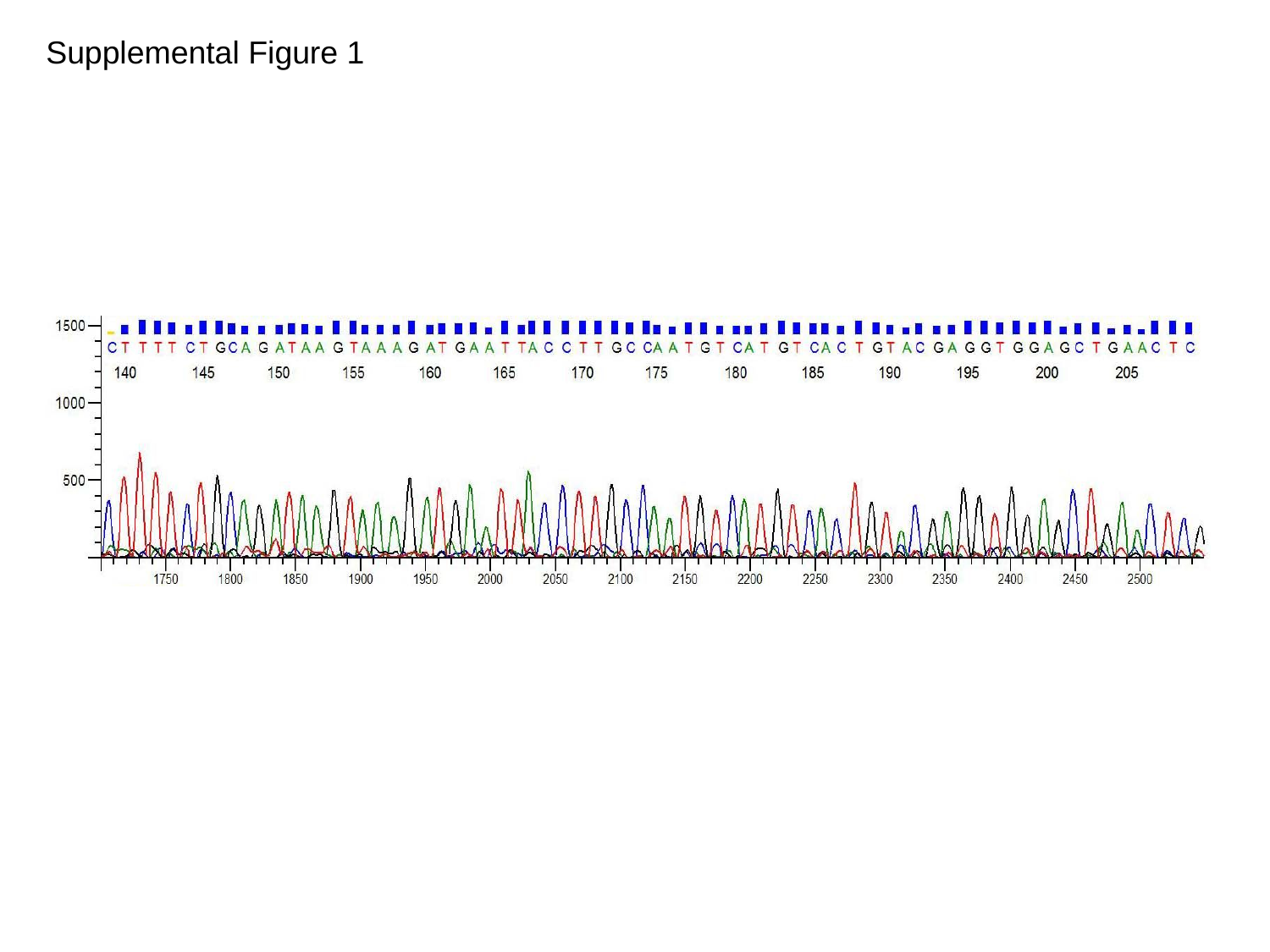

Supplemental Figure 1

Supplement: Supplementary Figure 1 [file 6605085x1.ppt]

## Slide 1
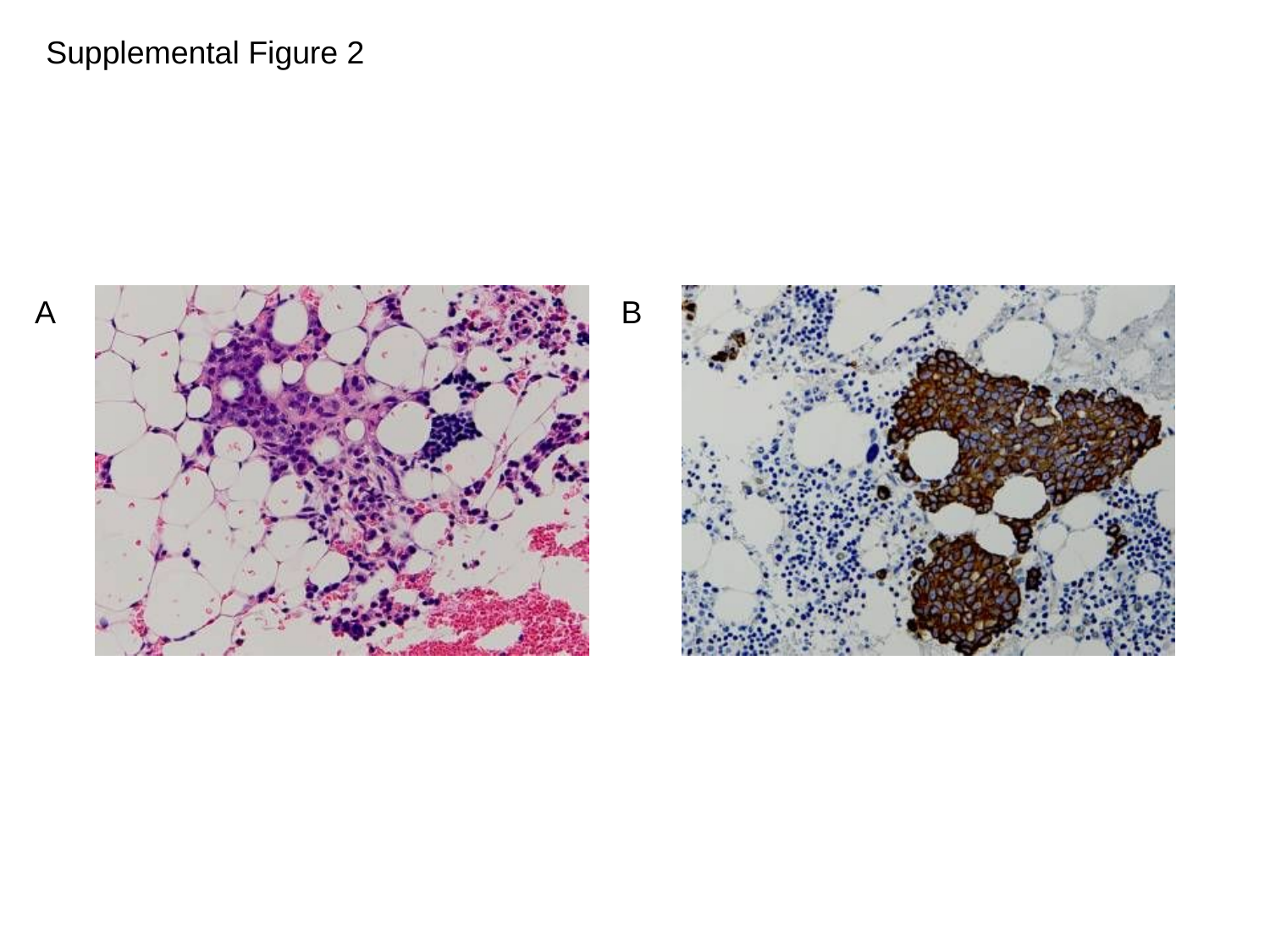

Supplemental Figure 2
A
B

Supplement: Supplementary Figure 2 [file 6605085x2.ppt]

## Slide 1
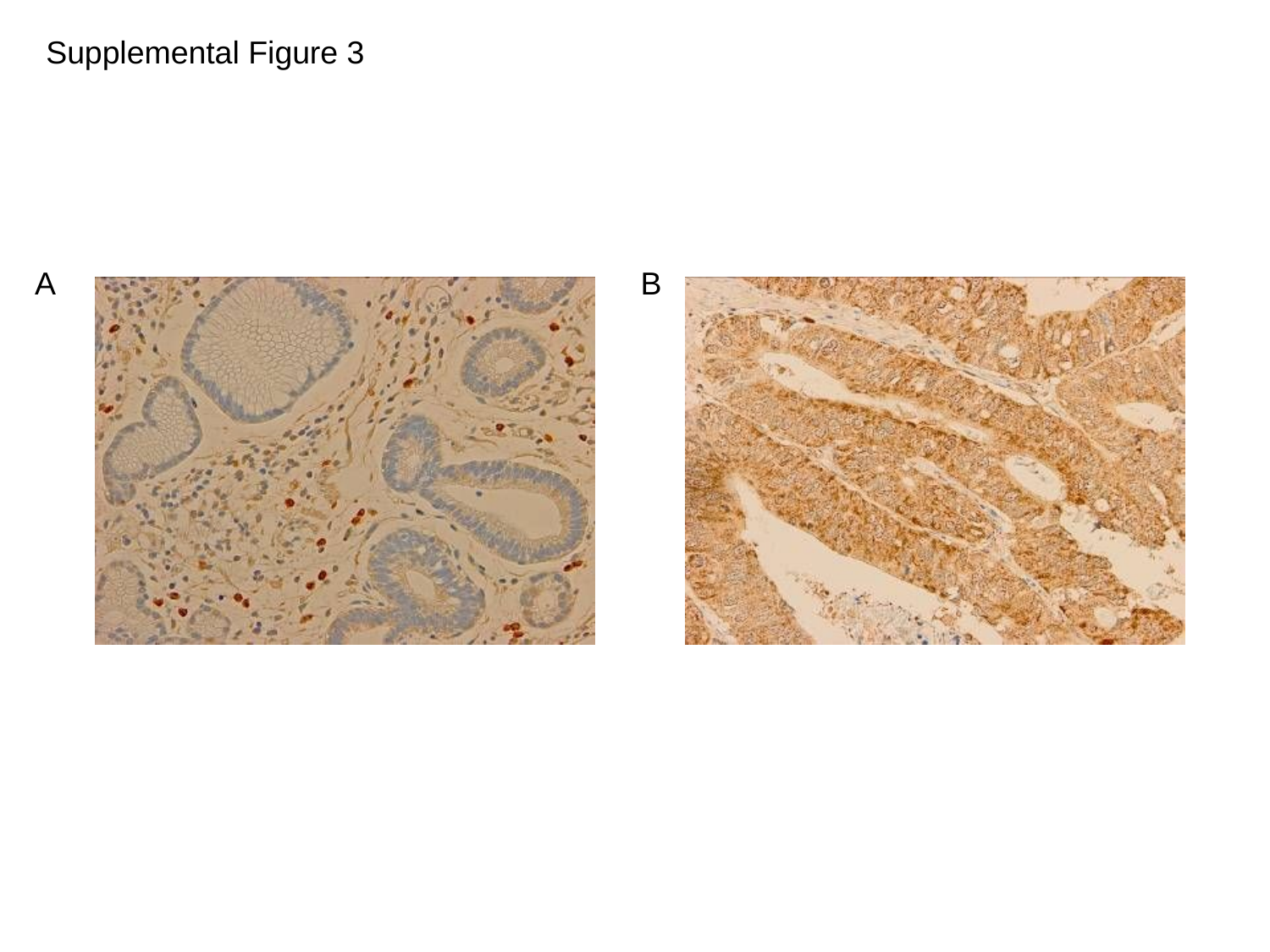

Supplemental Figure 3
A
B

Supplement: Supplementary Figure 3 [file 6605085x3.ppt]

## Slide 1
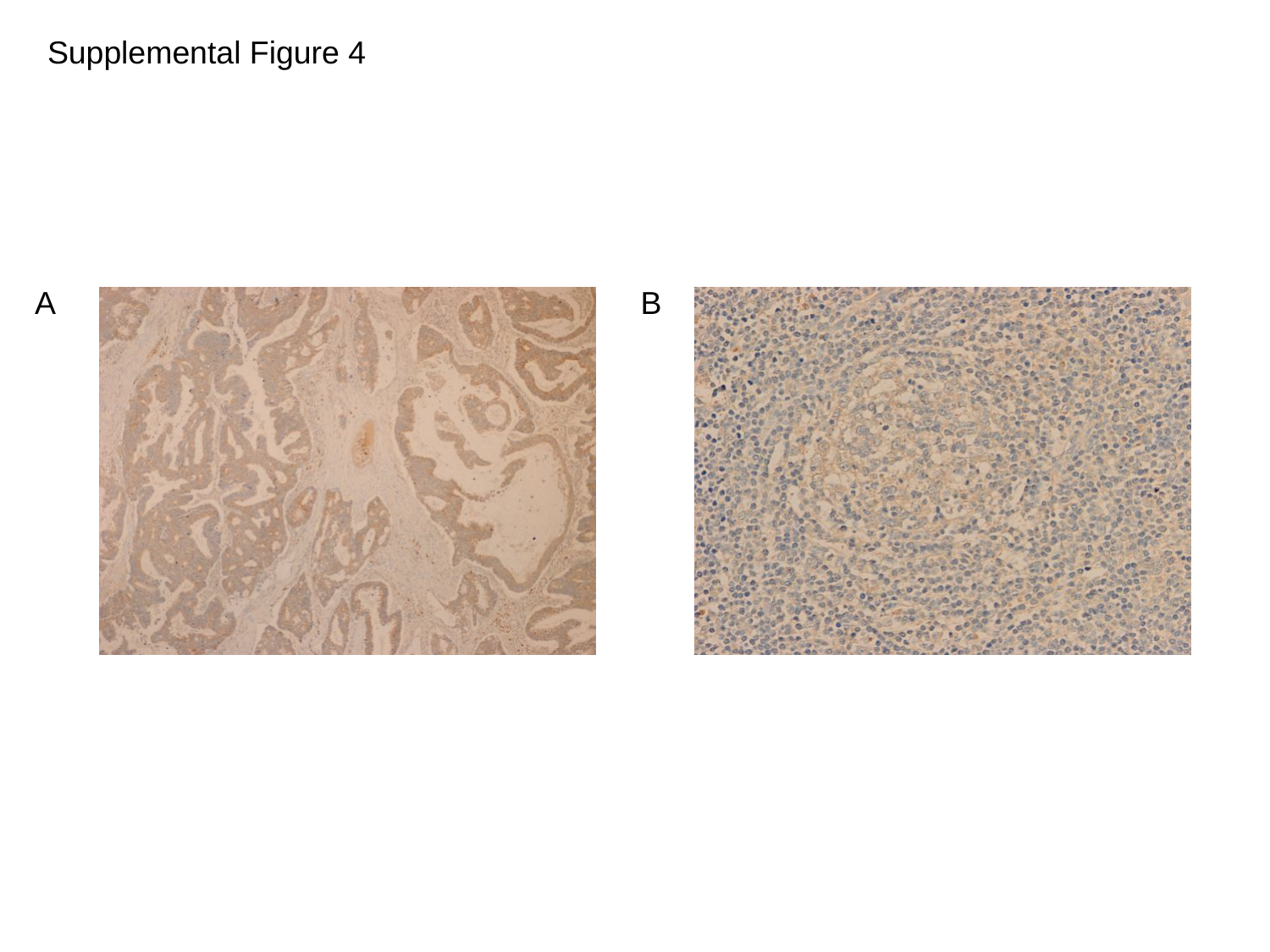

Supplemental Figure 4
A
B

Supplement: Supplementary Figure 4 [file 6605085x4.ppt]
