# Supplementary material for: Bone marrow and peripheral blood expression of ID1 in human gastric carcinoma patients is a bona fide indicator of lymph node and peritoneal metastasis
Source: Br J Cancer. 2009 Jun 2;100(12):1937–42. doi: 10.1038/sj.bjc.6605085 (PMC2714249; doi:10.1038/sj.bjc.6605085)
Supplement: Supplementary Figure Legends [file 6605085x5.doc]

**Figure Legends**

**Supplemental Figure 1.** The confirmation of the specificity of ID1 gene. The part of *ID1* confirmed by sequencing was shown.

**Supplemental Figure 2.** The case of bone marrow carcinomatosis resulting from metastasized gastric cancer was confirmed to be epithelial cells by HE (panel **A**) stain and AE1/AE3 (panel **B**). (**A, B:** original magnification:×100)

**Supplemental Figure 3.** The ID1 expression in primary lesion of gastric cancer cases. Two-thirds cases of primary lesions were stained strongly with ID1 antibody. Some of cases showed weak (panel A) or moderate (panel B) ID1 staining. (**A, B:** original magnification:×100)

**Supplemental Figure 4.** Negative control using the ID1 blocking peptide. Panel A (primary lesion) and panel B (metastatic lymph node lesion) showed ID1 negative expression by using the adjacent section stained with anti-ID1 antibody in Figure 5A and 5B, respectively (original magnification: **A:**×40, **B:**×100).
